# Association Between Residual Urine Volume and Recurrence Among Patients at High Risk of Non-Muscle-Invasive Bladder Carcinoma With Versus Without Bacillus Calmette-Guérin Treatment

**DOI:** 10.7759/cureus.61345

**Published:** 2024-05-30

**Authors:** Yuki Murakami, Tomokazu Sazuka, Ryo Tsukamoto, Hiroaki Sato, Keisuke Ando, Manato Kanesaka, Yasutaka Yamada, Yusuke Imamura, Shinichi Sakamoto, Tomohiko Ichikawa

**Affiliations:** 1 Urology, Chiba University Graduate School of Medicine, Chiba, JPN

**Keywords:** risk factor, non-muscle-invasive bladder cancer, intravesical recurrence, bacillus calmette-guérin (bcg), residual urine

## Abstract

Non-muscle-invasive bladder carcinoma often occurs in older adults, who often also have urinary dysfunction. The residual urine volume is an important indicator of urinary dysfunction. However, the impact of the residual urine volume on intravesical recurrence remains unclear. In the present study, we analyzed the data of 372 patients at high or very high risk of cancer progression according to the Japanese Urological Association classification who had undergone transurethral resection of a bladder tumor. In univariate analysis, postoperative absence of intravesical Bacillus Calmette-Guérin (BCG) induction was an independent risk factor for intravesical recurrence (hazard ratio 1.94, absence versus presence, p = 0.0019). The incidence of intravesical recurrence did not significantly differ between the mild, intermediate, and severe residual urine groups in the total cohort. Among the BCG-treated cohort, the three groups showed similar trends. Among the non-BCG-treated cohort, although the patients with more than 100 ml of residual urine tended to have more intravesical recurrence than patients with a smaller residual urine volume, this difference did not reach statistical significance. BCG treatment is recommended for patients at high risk of bladder carcinoma. Patients with a large residual urine volume without BCG treatment may be at high risk of intravesical recurrence.

## Introduction

Bladder cancer (BC) is the 10th most commonly diagnosed cancer worldwide [1]. About 70% of patients with BC are classified as having non-muscle-invasive urothelial cancer (NMIBC): some of these lesions progress to invasive cancer, which can be life-threatening, while some of these lesions cause recurrence, which impairs the patient’s quality of life due to repeated treatments [2,3]. Past reports and risk classifications have revealed various tumor-derived or adjuvant treatment-derived factors that affect recurrence [4]. However, the risk factors for intravesical recurrence associated with aging, including urinary status, have not been clarified. Recently, advanced age has been associated with inferior oncological outcomes [1,5]. Additionally, patients with a higher Charlson-Deyo Comorbidity Index have an increased risk of BC recurrence [6]. Such recurrence causes both a physical and an economic burden [7].

NMIBC affects many older adults, who often also have urinary dysfunction [7-9]. It is also predicted that urinary dysfunction will occur or worsen due to repeated transurethral resection (TUR) or intravesical injection therapy [10]. The presence of residual urine is used as an indicator of urinary dysfunction in daily clinical practice. Residual urine measurement is a simple and minimally invasive test that can be performed to identify urinary disorders in daily practice. We previously published a report on residual urine volume and recurrence in patients with NMIBC and upper urinary tract urothelial carcinoma [11,12]. Our previous study on NMIBC included patients with all levels of risk for NMIBC, including high-risk patients who are recommended to receive intravesical Bacillus Calmette-Guérin (BCG) injection and low-risk patients who should not receive intravesical BCG injection. This previous study showed that the presence of residual urine may be a risk factor for intravesical recurrence in patients with all levels of NMIBC risk. The presence of pyuria is also an independent risk factor for recurrence [12,13]. Therefore, in patients with mucous membranes that are inflamed before TUR, a constant state of residual urine volume that persists after TUR may promote the engraftment of BC cells after surgery, resulting in a higher recurrence rate.

Intravesical BCG injection therapy has a very strong prognostic effect on the recurrence of NMIBC [14]. However, even in situations where the risk classification indicates that intravesical BCG injection therapy should be performed, there are cases in which this treatment is not possible. Approximately 75% of Japanese patients who undergo intravesical BCG treatment receive a guideline-compliant induction regimen [15], which shows that not all patients who should undergo BCG treatment actually receive it. Even after risk classification, the presence or absence of BCG treatment has a significant impact on the outcome of recurrence.

The present study examined the impact of intravesical recurrence on the presence and amount of residual urine among patients who should initially have received intravesical BCG injection therapy (i.e., those with a Japanese Urological Association (JUA) classification of high risk or higher risk or cancer progression) and either did or did not receive BCG treatment. This information is important because high-risk groups are often difficult to treat. Furthermore, we believe that it is necessary to analyze NMIBC cases separately, as this is a heterogeneous group with different treatment intensities.

## Materials and methods

In the present study, conducted at Chiba University Graduate School of Medicine, Chiba, Japan, we analyzed the data of patients who had undergone TUR of a bladder tumor (TUR-Bt) for NMIBC between November 2008 and March 2022. The primary outcome was intravesical recurrence after TUR-Bt. This study was approved by the institutional review board of Chiba University (approval number 2554).

We classified each patient’s risk of cancer progression according to the JUA guidelines for NMIBC [16]. We excluded patients with pathologically identified muscle-invasive bladder carcinoma, those with a low or intermediate risk according to the JUA guidelines, and those with tumors other than bladder carcinoma. High risk is defined by the JUA as pT1 and/or carcinoma in situ (CIS) and/or high-grade disease.

In our hospital, preventive intravesical therapy with an anticancer agent is administered postoperatively to all patients, unless this treatment is omitted at the surgeon’s discretion. Additionally, TUR is performed for all patients with T1 and/or high-grade disease. Intravesical BCG induction therapy is performed in intermediate- and high-risk patients, and maintenance BCG intravesical therapy is performed in high-risk patients with two of the three high-risk factors (T1, CIS, and high-grade disease).

The following data were retrospectively collected from the hospital records: age, sex, preoperative pyuria, preoperative urine cytology, history of NMIBC, tumor grade, pathological T stage, concomitant CIS, and postoperative intravesical BCG induction. BCG treatment was defined as having been administered even if it had only been administered once as induction or maintenance. Severe residual urine was defined as ≥100 ml, intermediate residual urine was defined as 50-100 ml, and mild residual urine was defined as <50 ml. Residual urine measurement was performed three times, including before TUR-Bt and after surgery. The median value was used as the index. Patients were asked to urinate immediately before cystoscopy, and the residual urine was measured when the endoscope was inserted. Pyuria was defined as ≥10 white blood cells per high-powered field prior to TUR-Bt. Antibiotics were administered preoperatively to those with symptomatic urinary tract infections but not to patients with asymptomatic pyuria. On the day of TUR-Bt, all patients (with and without preoperative pyuria) received one dose of intravenous antibiotics. Positive preoperative urine cytology was defined as “positive” or class 4 or 5. Recurrence was defined as a diagnosis of intravesical bladder carcinoma or metastasis. Intravesical recurrence was diagnosed by cystoscopy, TUR, urine cytology, or computed tomography.

All statistical analyses were conducted using JMP Pro 16.0.1 (SAS Institute, Cary, North Carolina, United States). A two-sided p-value of <0.05 was considered to denote statistical significance. Comparisons were carried out using the Mann-Whitney and χ2 tests. Comparisons between the three groups were done using the Bonferroni method, and significant differences were defined as one-third of 0.05 between each of the two groups. Risk factors related to intravesical recurrence were analyzed by the Cox proportional hazards model and the Kaplan-Meier method. Factors with a p-value of 0.05 or less in the univariate analysis were included in the multivariate analysis.

## Results

**Figure 1:**
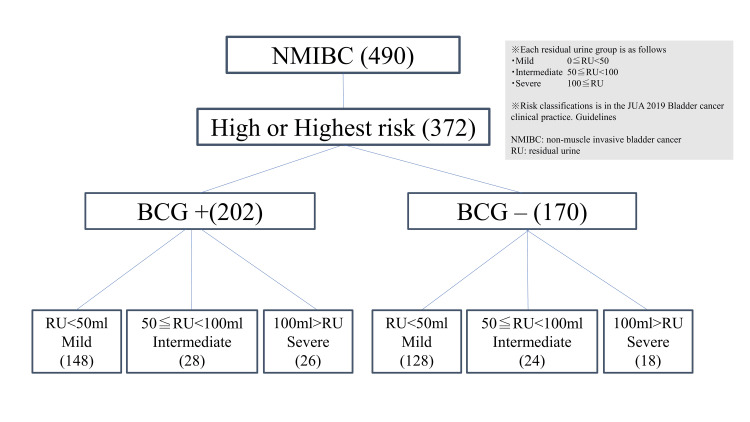
Flowchart of the inclusion, exclusion, and grouping of patients based on the presence or absence of BCG treatment and the RU volume BCG, Bacillus Calmette-Guérin; JUA, Japanese Urological Association; NMIBC, non-muscle-invasive urothelial cancer; RU, residual urine

During the study period, we treated 490 patients, 118 of whom were excluded because they had a JUA risk of low or intermediate; therefore, 372 eligible patients were included in the study. There were 202 patients who had received BCG treatment and 170 who had not. The flowchart of this study is shown in Figure 1. Among those who received BCG treatment, 26 had severe residual urine, 28 had intermediate residual urine, and 148 had mild residual urine. Among those who did not receive BCG treatment, 18 had severe residual urine, 24 had intermediate residual urine, and 128 had mild residual urine (Figure 1).

The median duration of follow-up for all 372 patients was 35.7 months. During the study period, 88 BC recurrence events occurred. Table 1 shows the background characteristics of the total cohort and of the groups classified by the amount of residual urine. The Bonferroni method revealed significant intergroup differences only regarding sex. The other clinical and pathological factors did not differ between the groups classified by the amount of residual urine.

**Table 1:** Patients’ characteristics BCG, Bacillus Calmette-Guerin; CIS, carcinoma in situ; NS, not significant; UUTUC, upper urinary tract urothelial carcinoma

		All (n = 372)	① Residual urine <50 ml (n = 276)	② 50 ml ≦ residual urine <100 ml (n = 52)	③ Residual urine ≧100ml (n = 44)	p-value
Median age (range)		73 (38-94)	38-94 (73)	51-84 (70)	73 (47-89)	NS
Sex						*Significant
	Male	289 (77.7%)	203 (73.6%)	46 (88.4%)	40 (90.9%)	*①:③p = 0.0124
	Female	83 (22.3%)	73 (26.4%)	6 (11.5%)	4 (9.1%)	
Grade						NS
	High	349 (93.8%)	261 (94.6%)	48 (92.3%)	40 (90.9%)	
	Low	23 (6.2%)	15 (5.4%)	4 (7.7%)	4 (9.1%)	
BCG						NS
	Present	202 (54.3%)	148 (53.6%)	28 (53.8%)	26 (59.1%)	
	Absent	170 (45.7%)	128 (46.4%)	24 (46.2%)	18 (40.9%)	
CIS						NS
	Present	55 (14.8%)	45 (16.3%)	7 (13.5%)	3 (6.8%)	
	Absent	317 (85.2%)	231 (83.7%)	45 (86.5%)	41 (93.2%)	
Pyuria						NS
	Present (≧10/HPF)	83 (22.3%)	58 (21.0%)	13 (25.0%)	12 (27.3%)	
	Absent	289 (77.7%)	218 (79.0%)	39 (75.0%)	32 (72.7%)	
Cytodiagnosis						NS
	Positive (Class ≧Ⅳ）	283 (76.1%)	74 (26.8%)	9 (17.3%)	6 (13.6%)	
	Negative	89 (23.9%)	202 (73.2%)	43 (82.7%)	38 (86.4%)	
Previous UUTUC						NS
	Present	60 (16.1%)	45 (16.3%)	5 (9.6%)	10 (22.7%)	
	Absent	312 (83.9%)	231 (83.7%)	47 (90.4%)	34 (77.3%)	
Timing						NS
	Primary	264 (71.0%)	194 (70.3%)	43 (82.7%)	27 (61.4%)	
	Recurrent	108 (29.0%)	82 (29.7%)	9 (17.3%)	17 (38.6%)	
Tumor number						NS
	Solitary	114 (31.1%)	79 (29.0%)	20 (39.2%)	15 (34.1%)	
	Multiple	253 (68.9%)	193 (71.0%)	31 (60.8%)	29 (65.9%)	

**Figure 2:**
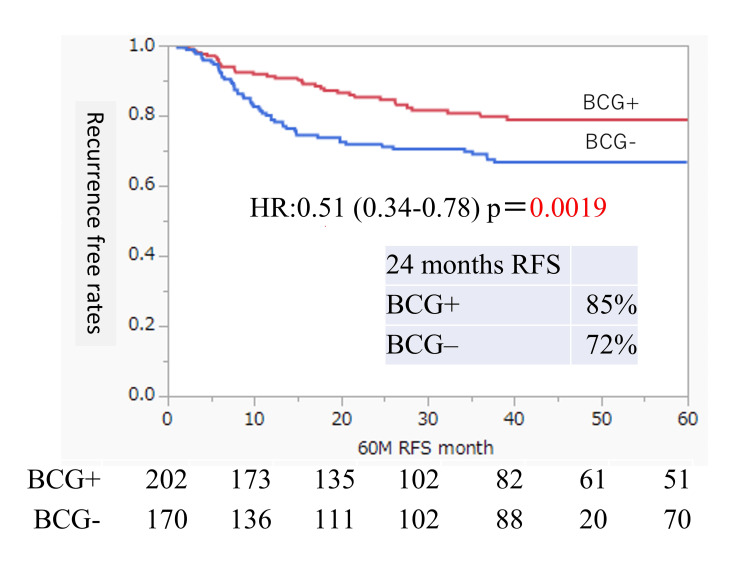
Intravesical RFS rates after TUR of bladder tumors according to the BCG treatment status Rates were estimated using the Kaplan-Meier method. BCG, Bacillus Calmette-Guérin; RFS, recurrence-free survival; TUR, transurethral resection

We examined the risk factors for postoperative BC recurrence in all 372 patients with the high or highest JUA risk classifications (Table 2). Univariate analysis showed that the only independent risk factor for intravesical recurrence was the postoperative absence of intravesical BCG induction (hazard ratio 1.94, absence versus presence, p = 0.0019). The recurrence-free survival (RFS) in the groups with and without intravesical BCG induction is shown in Figure 2.

**Table 2:** Risk factors for postoperative BC recurrence in patients with the high or highest JUA risk classification BC, bladder cancer; BCG, Bacillus Calmette-Guerin; CIS, carcinoma in situ; JUA, Japanese Urological Association; UUTUC, upper urinary tract urothelial carcinoma

		All (n = 372)	Univariate analysis HR (95% CI)	Univariate analysis p-value
Median age (range)		73 (38-94)		
Sex			1.18 (0.71-2.00)	0.5148
	Male	289 (77.7%)		
	Female	83 (22.3%)		
Grade			2.04 (0.65-6.45)	0.2247
	High	349 (93.8%)		
	Low	23 (6.2%)		
BCG			0.51 (0.34-0.78)	0.0019
	Present	202 (54.3%)		
	Absent	170 (45.7%)		
CIS			1.06 (0.61-1.84)	0.8428
	Present	55 (14.8%)		
	Absent	317 (85.2%)		
Pyuria			1.23 (0.77-1.97)	0.3888
	Present (≧10/HPF)	83 (22.3%)		
	Absent	289 (77.7%)		
Cytology diagnosis			1.19 (0.75-1.90)	0.461
	Positive (Class ≧Ⅳ)	283 (76.1%)		
	Negative	89 (23.9%)		
Previous UUTUC			1.59 (0.97-2.61)	0.0657
	Present	60 (16.1%)		
	Absent	312 (83.9%)		
Timing			0.84 (0.54-1.29)	0.4248
	Primary	264 (71.0%)		
	Recurrent	108 (29.0%)		
Tumor number			0.71 (0.44-1.15)	0.1606
	Solitary	114 (31.1%)		
	Multiple	253 (68.9%)		

**Figure 3:**
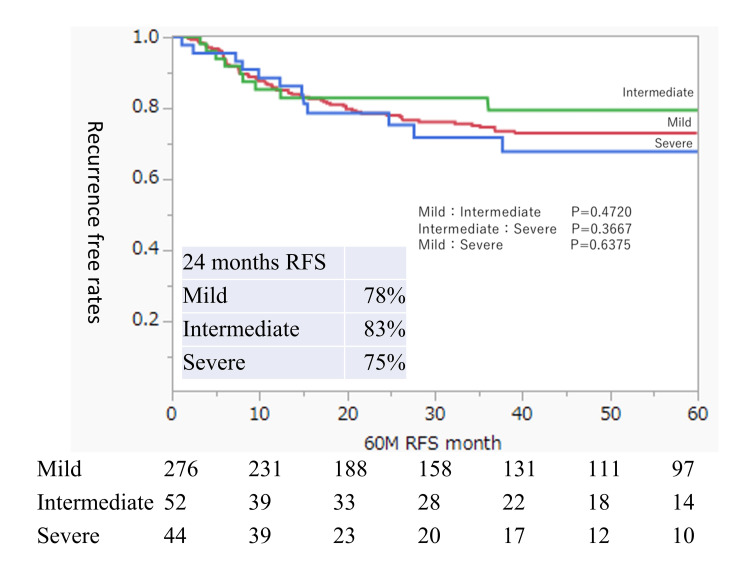
Intravesical RFS rates after TUR of bladder tumors in all patients according to the residual urine status Rates were estimated using the Kaplan-Meier method. The residual urine groups are defined as mild (0 ml ≤ residual urine volume < 50 ml), intermediate (50 ml ≤ residual urine volume < 100 ml), and severe (residual urine volume ≥ 100 ml). RFS, recurrence-free survival; TUR, transurethral resection

Next, we examined the RFS in the groups with mild, intermediate, and severe residual urine. This analysis included a mix of patients who did and did not receive BCG treatment. There were no significant differences in RFS between the three groups (Figure 3). The 24-month RFS rates were 78%, 83%, and 75% in the mild, intermediate, and severe residual urine groups, respectively.

**Figure 4:**
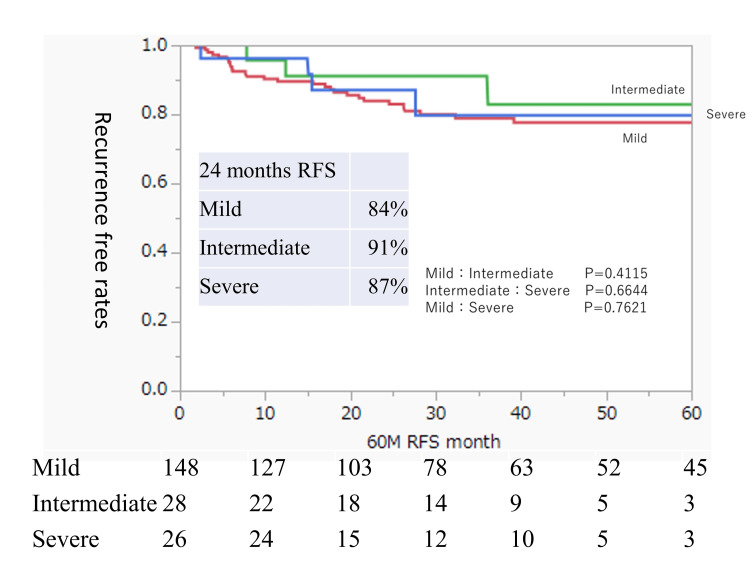
Intravesical RFS rates after TUR of bladder tumors in BCG-treated patients according to the residual urine status Rates were estimated using the Kaplan-Meier method. The residual urine groups are defined as mild (0 ml ≤ residual urine volume < 50 ml), intermediate (50 ml ≤ residual urine volume < 100 ml), and severe (residual urine volume ≥ 100 ml). BCG, Bacillus Calmette-Guerin; RFS, recurrence-free survival; TUR, transurethral resection

**Figure 5:**
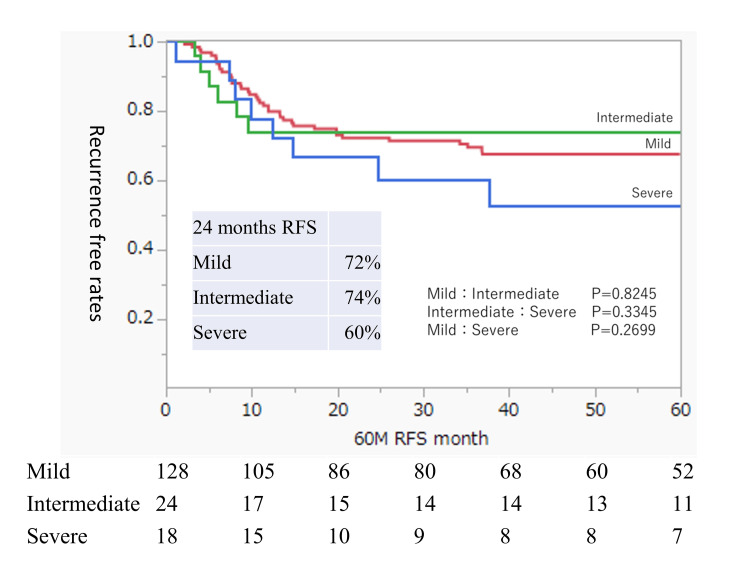
Intravesical RFS rates after TUR of bladder tumors in non-BCG-treated patients according to the residual urine status Rates were estimated using the Kaplan-Meier method. The residual urine groups are defined as mild (0 ml ≤ residual urine volume < 50 ml), intermediate (50 ml ≤ residual urine volume < 100 ml), and severe (residual urine volume ≥ 100 ml). Comparison of the three residual urine status groups. BCG, Bacillus Calmette-Guerin; RFS, recurrence-free survival; TUR, transurethral resection

We focused our analysis on residual urine by dividing the patients according to the presence or absence of intravesical BCG injection, which was the only risk factor for RFS identified in this cohort. Table 3 shows the background characteristics of the group that received BCG treatment (BCG group; n = 202). Table 4 shows the background characteristics of the group that did not receive BCG treatment (non-BCG group; n = 170). In the BCG group, there were significant differences between the residual urine groups in sex and the timing of bladder tumor occurrence (Table 3). In contrast, the non-BCG group had no significant differences in patient characteristics depending on the post-void residual urine volume (Table 4). Figure 4 shows the RFS analysis of the BCG group divided into three groups based on the residual urine volume; the three groups showed similar trends with no significant differences. Figure 5 shows the RFS analysis of the non-BCG group; there was no significant difference between the three groups, although the mild and intermediate residual urine groups showed similar trends while the RFS of the severe residual urine group tended to be lower. In the non-BCG group, the 24-month RFS rates were 72%, 74%, and 60% in the mild, intermediate, and severe residual urine groups, respectively.

**Table 3:** Background characteristics of the patients with BCG treatment *①:③p = 0.0160; **①:②p = 0.0081 BCG, Bacillus Calmette-Guerin; CIS, carcinoma in situ; NS, not significant; UUTUC, upper urinary tract urothelial carcinoma

		All (n = 202)	① Residual urine <50 ml (n = 148)	② 50 ml ≦ residual urine <100 ml (n = 28)	③ Residual urine ≧100ml (n = 26)	p-value
Median age (range)		73 (38-89)	74 (38-89)	69.5 (54-84)	75.5 (47-87)	
Sex						*Significant
	Male	160 (79.2%)	111 (75.0%)	24 (85.7%)	25 (96.2%)	
	Female	42 (20.8%)	37 (25.0%)	4 (14.3%)	1 (3.8%)	
Grade						NS
	High	181 (89.6%)	134 (90.5%)	25 (89.3%)	22 (84.6%)	
	Low	21 (10.4%)	14 (9.5%)	3 (10.7%)	4 (15.4%)	
CIS						NS
	Present	53 (26.2%)	30 (20.3%)	4 (14.3%)	8 (30.8%)	
	Absent	149 (73.8%)	118 (79.7%)	24 (85.7%)	18 (69.2%)	
Pyuria						NS
	Present (≧10/HPF)	61 (30.2%)	36 (24.3%)	9 (32.1%)	3 (11.5%)	
	Absent	141 (69.8%)	112 (75.7%)	19 (67.9%)	23 (88.5%)	
Cytodiagnosis						NS
	Positive (Class ≧Ⅳ）	24 (11.9%)	52 (35.1%)	6 (21.4%)	3 (11.5%)	
	Negative	178 (88.1%)	96 (64.9%)	22 (78.6%)	23 (88.5%)	
Previous UUTUC						NS
	Present	144 (71.3%)	19 (12.8%)	2 (7.1%)	15 (57.7%)	
	Absent	58 (28.7%)	129 (87.2%)	26 (92.9%)	11 (42.3%)	
Timing						**Significant
	Primary	147 (73.9%)	104 (70.3%)	25 (89.3%)	18 (69.2%)	
	Recurrent	52 (26.1%)	44 (29.7%)	3 (10.7%)	8 (30.8%)	
Tumor number						NS
	Solitary	131 (64.9%)	34 (23.4%)	10 (35.7%)	21 (80.8%)	
	Multiple	71 (35.2%)	111 (76.6%)	18 (64.3%)	5 (19.2%)	

**Table 4:** Background characteristics of the patients without BCG treatment BCG, Bacillus Calmette-Guerin; CIS, carcinoma in situ; NS, not significant; UUTUC, upper urinary tract urothelial carcinoma

		All (n = 170)	① Residual urine <50 ml (n = 128)	② 50 ml ≦ residual urine <100 ml (n = 24)	③ Residual urine ≧100 ml (n = 18)	p-value
Median age (range)		73 (39-94)	72 (39-94)	73 (51-84)	75.5 (56-89)	
Sex						NS
	Male	129 (75.9%)	92 (71.9%)	22 (91.7%)	15 (83.3%)	
	Female	41 (24.1%)	36 (28.1%)	2 (8.3%)	3 (16.7%)	
Grade						NS
	High	168 (98.8%)	127 (99.2%)	23 (95.8%)	18 (100%)	
	Low	2 (1.2%)	1 (0.8%)	1 (4.2%)	0 (0%)	
CIS						NS
	Present	30 (17.7%)	15 (11.7%)	3 (12.5%)	4 (22.2%)	
	Absent	140 (82.3%)	113 (88.3%)	21 (87.5%)	14 (77.8%)	
Pyuria						NS
	Present (≧10/HPF)	28 (16.5%)	22 (17.2%)	4 (16.7%)	3 (16.7%)	
	Absent	142 (83.5%)	106 (82.8%)	20 (83.3%)	15 (83.3%)	
Cytodiagnosis						NS
	Positive (Class ≧Ⅳ）	36 (21.25)	22 (17.2%)	3 (12.5%)	7 (38.9%)	
	Negative	134 (78.8%)	106 (82.8%)	21 (87.5%)	11 (61.1%)	
Previous UUTUC						NS
	Present	120 (70.6%)	26 (20.3%)	3 (12.5%)	12 (66.7%)	
	Absent	50 (29.4%)	102 (79.7%)	21 (87.5%)	6 (33.3%)	
Timing						NS
	Primary	106 (63.1%)	90 (70.3%)	18 (75.0%)	11 (61.1%)	
	Recurrent	62 (36.9%)	38 (29.7%)	6 (25.0%)	7 (38.9%)	
Tumor number						NS
	Solitary	125 (73.5%)	45 (35.4%)	10 (43.5%)	13 (72.2%)	
	Multiple	45 (26.5%)	82 (64.6%)	13 (56.5%)	5 (27.8%)	

**Figure 6:**
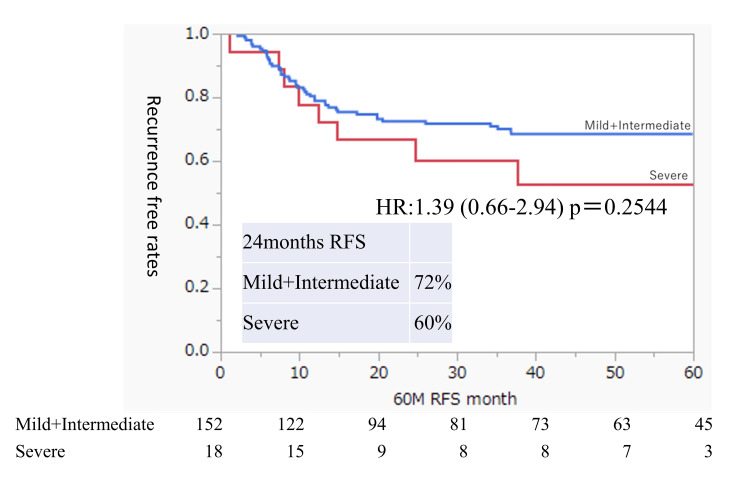
Intravesical RFS rates after TUR of bladder tumors in non-BCG-treated patients according to the residual urine status Rates were estimated using the Kaplan-Meier method. The residual urine groups are defined as mild (0 ml ≤ residual urine volume < 50 ml), intermediate (50 ml ≤ residual urine volume < 100 ml), and severe (residual urine volume ≥ 100 ml). Comparison of the two residual urine status groups (mild plus intermediate versus severe). BCG, Bacillus Calmette-Guerin; RFS, recurrence-free survival; TUR, transurethral resection

Figure 6 shows the RFS compared between the severe residual urine group and the combined mild and intermediate residual urine groups. Although the number of patients in the severe group was small and there was no significant difference in RFS between the two groups (p = 0.2544), having severe residual urine in the non-BCG group may be a risk factor for intravesical recurrence.

Our results showed that the RFS was significantly worse in high-risk or higher-risk patients who could not receive BCG treatment. Among the non-BCG group, patients with severe residual urine tended to have poor RFS. BCG administration may offset the risk factors for residual urine.

## Discussion

The current real-world study of patients with a high or higher risk of cancer progression showed that the RFS was significantly worse in patients who did not receive BCG treatment compared with those who did receive BCG treatment. The American Urological Association and European Association of Urology guidelines recommend intravesical BCG therapy for patients with a high or higher risk of cancer progression and recommend maintenance BCG therapy in addition to induction therapy [17,18]. However, there are cases in which appropriate BCG treatment cannot be performed due to poor urinary conditions after TUR-Bt or other systemic diseases. Adverse events following intravesical BCG therapy are related to strain virulence, allergic reactions, or nosocomial urinary tract infections [19]. Furthermore, patients with persistent hematuria have an increased risk of systemic dissemination of Mycobacterium tuberculosis due to BCG treatment [20]. The performance of widespread TUR and second TUR can also lead to prolonged urination frequency and pain during urination. Indeed, our results indicate that a wide range of patients (such as those mentioned above or those who have undergone repeated TUR) were not able to receive the recommended BCG treatment. It is possible that there was an unavoidable bias in our study because the data were obtained from actual clinical practice. However, our results showed that patients who are eligible for BCG treatment should receive BCG treatment to improve their RFS.

The urinary status of patients with NMIBC has important implications for maintaining quality of life through stable urination and the administration of appropriate BCG treatment [10,21,22]. The quality of life of patients with non-muscle-invasive BC shows significant improvement in the fourth week after BCG induction [23]. The guidelines and recommendations on the management of lower urinary tract symptoms and urinary incontinence recommend measurement of the post-void residual urine volume [24-26]. Therefore, we compared the RFS between three groups classified based on the amount of residual urine (mild, intermediate, and severe). Residual urine volume is easily measured in daily clinical practice and is a reliable objective outcome measure [27,28]. Considering the possibility that the true residual urine volume may not be accurately evaluated with a single measurement, the median value of three measurements was defined as the residual urine volume of each patient in the present study. In our previous report, even after background matching for all risk factors, patients with a large amount of residual urine had poor RFS [12]. The present study cohort was limited to patients with a JUA classification of high risk or higher; thus, the nature of the original cancer was worse in the present cohort than in the previous cohort [12]. The previous and present findings suggest that the impact of residual urine may be more pronounced in patients with a lower JUA risk classification than in those with a high JUA risk classification. We will accumulate more cases and investigate this further in a future study. The present study showed no difference in RFS based on the residual urine volume, regardless of whether BCG treatment was performed. Similarly, there was no difference in residual urine volume between the BCG and non-BCG groups. Among the cases in which BCG could not be performed, the RFS of patients with a residual urine volume of 100 ml or more tended to be poorer compared with patients with mild or intermediate residual urine volumes, although these differences did not reach statistical significance. The limited number of patients made it difficult to detect statistically significant differences. However, these results suggest that the impact of residual urine volume on RFS may change depending on the presence or absence of BCG treatment, a factor that affects RFS.

We believe that the residual urine affects RFS via the following mechanism: patients with a large residual urine volume may have cancer present in the bladder before TUR or may have a large number of cancer cells floating in the bladder immediately after TUR; therefore, they may have more BC cells exposed to the bladder mucosa for a longer period of time compared with patients with a small residual urine volume. As a result, we speculate that tumor dissemination is more likely to occur within the bladder of patients with a large residual urine volume than in those with a small residual urine volume. The presence of pyuria is also reportedly an independent risk factor for poor RFS in patients with BC [13,29,30]. Furthermore, prolonged exposure of the mucous membranes to urine-containing BC cells is thought to cause inflammation and may promote dissemination. However, no large-scale prospective trials have investigated this issue.

The present study has some limitations. First, this study was a retrospective study. According to the guidelines, all patients in the present study cohort should have undergone BCG treatment; however, the non-BCG group comprised patients who could not receive BCG treatment due to certain circumstances. Additionally, most patients were in the mild residual urine group, while there were few patients in the severe residual urine group. Thus, there was a wide range in the number of patients between the groups being compared, and the present findings require confirmation in a study with a larger number of patients. Finally, we have not been able to evaluate the interleukin assay or T cell count after BCG, which is a future topic.

The measurement of the post-void residual urine volume is a minimally invasive testing method that can be easily used in daily clinical practice. The present study was the first to focus on patients at high or highest risk of NMIBC and evaluate the influence of residual urine volume on the RFS depending on the presence or absence of BCG treatment. BCG treatment significantly improved the RFS in the group at high or highest risk of NMIBC. Furthermore, in the cohort in which BCG treatment was not possible, a residual urine volume of 100 ml or more may have increased the risk of recurrence.

## Conclusions

The present study was the first to focus on patients at high or highest risk of NMIBC and evaluate the influence of residual urine volume on the RFS depending on the presence or absence of BCG treatment. Our findings suggest that a residual urine volume of 100 ml or more may be a risk factor for postoperative recurrence of NMIBC in patients without BCG treatment, although we did not detect a statistically significant difference in RFS between groups classified based on the residual urine volume. Validation in larger prospective studies is required.
